# Genomic Epidemiology of Multidrug‐Resistant Clinical *Klebsiella pneumoniae* From Pakistan

**DOI:** 10.1002/mbo3.70381

**Published:** 2026-08-23

**Authors:** Hakeem Zada, Muhammad Qasim, Mubashir Hussain, Abdul Rehman, Hassan Naveed, Noor Bahadar, Najibullah Anwari

**Affiliations:** ^1^ Department of Microbiology Kohat University of Science and Technology Kohat Pakistan; ^2^ Transgenic Research Center, School of Life Sciences Northeast Normal University Changchun Jilin China; ^3^ Information Communication Technology Department Kabul University of Medical Sciences Kabul Afghanistan

**Keywords:** antimicrobial resistance, genomic epidemiology, *Klebsiella pneumoniae*, NDM‐1, plasmid replicons, single‐nucleotide polymorphisms

## Abstract

**Background:**

Antimicrobial‐resistant *Klebsiella pneumoniae* is a major clinical threat in Pakistan and the wider South Asian region, where genomic surveillance remains limited. We analyzed 122 publicly available human clinical *K. pneumoniae* whole‐genome sequencing datasets from Pakistan to define lineage structure, antimicrobial‐resistance determinants, virulence‐associated loci, surface‐antigen profiles, plasmid replicons, and genomic relatedness.

**Methods:**

Reads were extracted, quality‐filtered and assembled de novo, and assembly quality was assessed. Assemblies were characterized using Kleborate and ABRicate with the PlasmidFinder database. Trimmed reads were mapped to the *K. pneumoniae* MGH 78578 reference genome, and high‐confidence biallelic SNPs (QUAL ≥ 30; per‐isolate depth ≥ 10) were used to calculate pairwise genomic distances.

**Results:**

The cohort contained 23 sequence types and was dominated by ST15 (41/122, 33.61%), followed by ST48 (17/122, 13.93%), ST336 (11/122, 9.02%), ST711 (7/122, 5.74%), and ST3805 (7/122, 5.74%). Acquired resistance profiling detected 48 distinct antimicrobial‐resistance genes. *bla*_CTX‐M‐15_ was present in 121 isolates (99.18%), *bla*_VEB‐5_ in 45 (36.89%), and *bla*_NDM‐1_ in 16 (13.11%). *bla*_NDM‐1_‐positive isolates occurred in four principal ST/capsule/O‐locus backgrounds. Yersiniabactin and aerobactin loci were detected in 48 (39.34%) and 43 (35.25%) isolates, respectively; 43 isolates had a Kleborate virulence score ≥ 3, whereas colibactin and *rmpADC* were not detected. IncFIB(K)_1_Kpn3 was the most frequent plasmid replicon marker (101/122, 82.79%). Analysis of 169,220 high‐confidence biallelic SNP sites yielded 7381 pairwise comparisons, with a median distance of 30,224 SNPs and no pairs differing by ≤ 10 SNPs.

**Conclusion:**

The cohort showed broad genomic diversity, near‐universal *bla*_CTX‐M‐15_ carriage, lineage‐structured *bla*_NDM‐1_ backgrounds, frequent siderophore‐associated virulence loci, and distinct plasmid‐replicon profiles. The findings support multiple co‐circulating multidrug‐resistant genomic backgrounds rather than a single clonal expansion and provide a reference point for genomic surveillance in Pakistan.

## Graphical abstract

Study design and bioinformatics workflow. Cohort selection and uniform workflow.

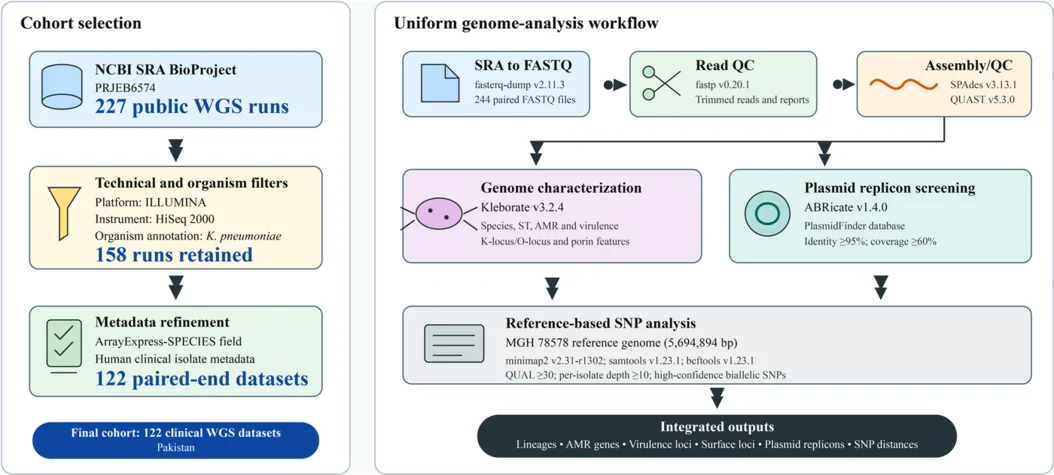

## 1 Introduction

Antimicrobial resistance (AMR) among Gram‐negative bacteria is a major public health threat because it compromises the treatment of severe infections. The 2024 World Health Organization bacterial priority pathogens list designates carbapenem‐resistant Enterobacterales and third‐generation cephalosporin‐resistant Enterobacterales as critical‐priority pathogens (World Health Organization 2024). *Klebsiella pneumoniae* is a prominent member of Enterobacterales and causes hospital‐ and community‐associated infections, including pneumonia, bloodstream infection, urinary tract infection, wound infection, and neonatal sepsis (Huang et al. 2025; Paczosa and Mecsas 2016).

The clinical success of *K. pneumoniae* is linked to genome plasticity and the capacity to acquire antimicrobial‐resistance determinants, mobile genetic elements, and virulence‐associated loci. Extended‐spectrum β‐lactamases (ESBLs), particularly CTX‐M‐type enzymes, have disseminated globally and compromise the activity of third‐generation cephalosporins (Lee et al. 2016; Bevan et al. 2017). Plasmid‐mediated quinolone‐resistance determinants further broaden resistance profiles (Jacoby et al. 2014). Carbapenemases, including NDM‐type metallo‐β‐lactamases, severely restrict treatment options and are especially relevant to South Asia (Lee et al. 2016; Walsh 2011). Alterations affecting the major porins OmpK35 and OmpK36 can further reduce β‐lactam permeability (Tsai et al. 2011; Sugawara et al. 2016).

Whole‐genome sequencing (WGS) enables simultaneous resolution of lineage structure, antimicrobial‐resistance determinants, virulence‐associated loci, surface‐antigen backgrounds, plasmid replicon markers, and genome‐wide relatedness. In *K. pneumoniae*, integration of sequence type, capsule and O‐antigen loci, resistance and virulence genotypes, and SNP‐based distances can identify clinically important genomic backgrounds while distinguishing broad population diversity from close within‐lineage relatedness (Mejía‐Limones et al. 2024; Punina et al. 2015; Lam et al. 2021).

Pakistan is situated in South Asia, where AMR among Enterobacterales is a major public health concern. Studies from Pakistan have documented multidrug‐resistant *K. pneumoniae*, ESBL production, and NDM‐associated carbapenem resistance, including plasmid‐mediated dissemination (Khan et al. 2025; Idrees et al. 2023; Qamar et al. 2025; Lascols et al. 2023). However, integrated genome‐scale analyses combining lineages, resistance, virulence, surface loci, plasmid replicons, and SNP‐based relatedness remain limited.

In this study, we analyzed 122 publicly available human clinical *K. pneumoniae* WGS datasets from Pakistan using a uniform analytical workflow. We defined sequence‐type composition, resistance and virulence‐associated features, capsule and O‐antigen loci, plasmid replicon profiles and reference‐based SNP relatedness to identify the principal lineage‐associated genomic backgrounds represented in the cohort.

## 2 Materials and Methods

### 2.1 Study Design and Isolate Selection

We performed a comparative genomic analysis of publicly available *K. pneumoniae* WGS data retrieved from the NCBI Sequence Read Archive (SRA). Candidate records were identified from BioProject PRJEB6574 using the NCBI SRA Run Selector. The initial BioProject query returned 227 public WGS runs. To obtain a technically consistent dataset, filters were applied for sequencing platform, instrument model and organism annotation, retaining runs generated on the ILLUMINA platform with the Illumina HiSeq. 2000 instrument and annotated as *K. pneumoniae* subsp. *pneumoniae*. These filters reduced the dataset to 158 runs. A final metadata refinement retained 122 runs for which the ArrayExpress‐SPECIES field was empty, corresponding to the target ERR‐series cohort. Accession‐level metadata recorded for retained runs included Run, BioSample, Experiment, and Study accessions; platform, instrument, and library layout; organism annotation; and host, isolation‐source, location, and collection‐date fields when available. All 122 retained paired‐end runs were included in downstream analyses.

### 2.2 Computational Environment, Data Management, and Read Processing

Analyses were performed on a Google Cloud Platform Compute Engine C4 high‐memory virtual machine (c4‐highmem‐48; 48 vCPUs, 372 GB RAM) running Ubuntu 22.04.5 LTS. Raw and processed data were stored in a regional Google Cloud Storage bucket, while active computations were performed on an attached Hyperdisk Balanced volume. Raw SRA files were converted to paired‐end FASTQ format using fasterq‐dump v2.11.3 with split‐pair output enabled and compressed with pigz v2.6. Read quality control and adapter/quality trimming were performed using fastp v0.20.1 in paired‐end mode with automatic adapter detection (Chen et al. 2018). Trimmed FASTQ files and HTML/JSON reports were generated for each isolate, and JSON metrics were aggregated to summarize pre‐ and post‐filtering read counts, base counts, Q20/Q30 metrics, GC content, and duplication rate.

### 2.3 De Novo Assembly and Assembly Quality Assessment

Trimmed paired‐end reads were assembled de novo using SPAdes v3.13.1 with the—careful option (Bankevich et al. 2012). Assembly quality was evaluated using QUAST v5.3.0 (Gurevich et al. 2013). Metrics extracted for cohort‐level review included total contig count, the number of contigs ≥ 1000 bp, total assembly length, total length of contigs ≥ 1000 bp, largest contig length, GC content, N50, N75, L50, and L75. Successful assembly was confirmed by the presence of contigs.fasta, and all 122 isolates generated final assemblies and valid QUAST reports.

### 2.4 Lineage, Resistance, Virulence, Surface‐Antigen, and Plasmid Replicon Profiling

Genome‐based characterization was performed using the final assemblies. Species confirmation, multilocus sequence typing, acquired antimicrobial‐resistance determinants, chromosomal resistance‐associated mutations, virulence loci, Kleborate virulence score, *wzi* allele, and capsule and O‐antigen loci were assessed using Kleborate v3.2.4 with the *K. pneumoniae* species‐complex preset (Lam et al. 2021). For descriptive analyses, a Kleborate virulence score ≥ 3 was used to identify isolates with higher‐score genomic virulence profiles; this score was not interpreted as proof of a hypervirulent phenotype. Plasmid replicon screening was performed with ABRicate v1.4.0 and the PlasmidFinder database distributed with ABRicate (488 nucleotide sequences; database update April 3, 2026) (Carattoli et al. 2014), using minimum identity and coverage thresholds of 95% and 60%, respectively. Replicon calls were summarized at isolate and sequence‐type levels and interpreted as replicon markers rather than resolved plasmid structures or direct evidence of gene‐replicon linkage.

### 2.5 Reference Preparation, Read Mapping, and Per‐Isolate Variant Calling

Reference‐based analyses used the *K. pneumoniae* MGH 78578 genome assembly (RefSeq GCF_000016305.1; six sequences; 5,694,894 bp). The reference FASTA was indexed using samtools faidx v1.23.1 and minimap2 v2.31‐r1302 (Li 2018). Trimmed paired‐end reads were mapped independently in minimap2 short‐read mode, and alignments were coordinate‐sorted and indexed with samtools. Mapping summaries were generated using samtools flagstat and samtools coverage. Per‐isolate variant calling was performed using bcftools v1.23.1 (mpileup followed by call). High‐confidence variant sets retained sites with QUAL ≥ 30 and per‐isolate depth ≥ 10.

### 2.6 Cohort‐Level SNP Calling and Pairwise Genomic‐Distance Analysis

A cohort‐level SNP dataset was generated from the 122 coordinate‐sorted BAM files using bcftools v1.23.1 with haploid ploidy settings. The cohort VCF was reheadered with isolate accession identifiers and filtered to retain high‐confidence biallelic SNPs with QUAL ≥ 30. Pairwise distances were calculated with custom Python scripts (Python v3.10.12). At each variable site in the cohort VCF, an isolate genotype was considered callable only when it was unambiguous and supported by a depth of ≥ 10; the pairwise distance was the number of discordant alleles across sites callable in both isolates. Thresholds of ≤ 10, ≤ 25, and ≤ 50 SNPs were used only as descriptive screening categories for increasingly close genomic relatedness, because SNP cutoffs are method‐ and context‐dependent and should not be interpreted as universal transmission thresholds (Luterbach et al. 2023). No formal transmission inference was attempted.

## 3 Results

### 3.1 Genome Dataset and Lineage Composition

**Figure 1:**
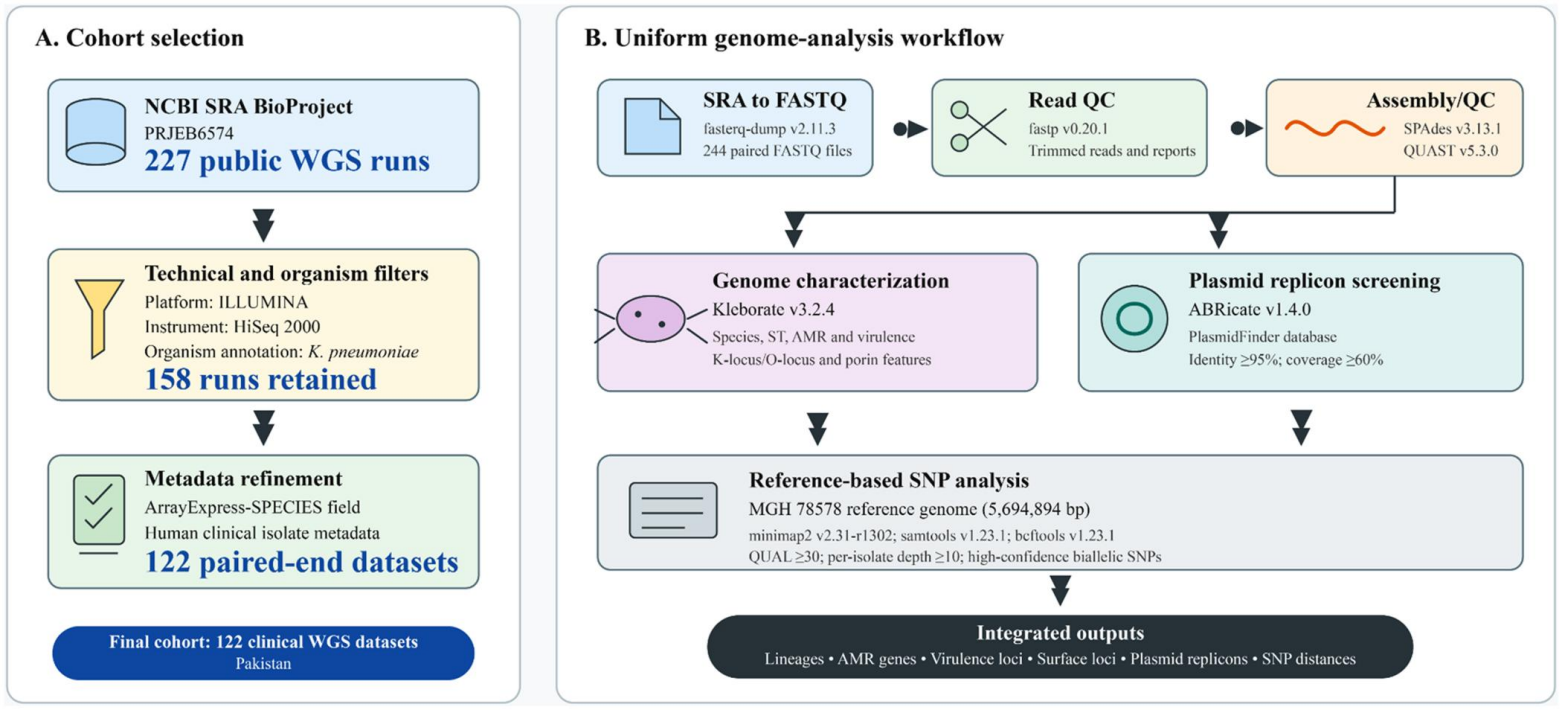
Study design and bioinformatics workflow. (A) Cohort selection from 227 public WGS runs in BioProject PRJEB6574 to 158 technically consistent runs and a final set of 122 paired‐end clinical datasets. (B) Uniform workflow for read processing, de novo assembly, and quality assessment, Kleborate‐based genome characterization, PlasmidFinder replicon screening, and reference‐based SNP analysis.

The study design and analysis workflow are summarized in Figure 1. The final dataset comprised 122 Illumina paired‐end WGS datasets assigned to *K. pneumoniae* subsp. *pneumoniae*. Genome‐based sequence typing identified 23 sequence types. ST15 was the most frequent lineage (41/122, 33.61%), followed by ST48 (17/122, 13.93%), ST336 (11/122, 9.02%), ST711 (7/122, 5.74%), and ST3805 (7/122, 5.74%). ST17, ST22, and ST38 were each represented by four isolates (3.28%), and the remaining sequence types occurred at lower frequencies. The population therefore comprised one dominant lineage and multiple recurrent or low‐frequency backgrounds rather than a single clonal expansion.

### 3.2 Antimicrobial‐Resistance Gene Landscape

Genome‐based profiling identified 48 distinct acquired antimicrobial‐resistance genes among the 122 isolates. *bla*_CTX‐M‐15_ was detected in 121 isolates (99.18%), *bla*_VEB‐5_ in 45 (36.89%), and *bla*_NDM‐1_ in 16 (13.11%). Plasmid‐mediated quinolone‐resistance determinants were detected in 109 isolates (89.34%), and Kleborate predicted porin alterations in 63 isolates (51.64%). These results indicate a near‐universal ESBL background with frequent additional quinolone‐ and permeability‐associated resistance features and a smaller carbapenemase‐positive subset (Table 1).

**Table 1:** Major antimicrobial‐resistance features detected in the 122‐isolate cohort.

AMR feature	Gene/class	Count	Percentage	Main interpretation
Total AMR genes detected	Acquired AMR gene repertoire	48 genes	—	Broad acquired resistance repertoire detected across the cohort
*bla*_CTX‐M‐15_	Extended‐spectrum β‐lactamase (ESBL)	121/122	99.18	Near‐universal ESBL determinant
*bla*_VEB‐5_	β‐lactamase	45/122	36.89	Frequent additional beta‐lactamase determinant
*bla*_NDM‐1_	Carbapenemase	16/122	13.11	Clinically important carbapenemase‐positive subset
PMQR determinants	Quinolone‐associated resistance	109/122	89.34	Widespread plasmid‐mediated quinolone‐resistance markers
Porin‐associated features	Permeability‐associated resistance	63/122	51.64	Frequent outer‐membrane resistance‐associated features

### 3.3 Carbapenemase‐Positive Genomic Backgrounds

Carbapenemase‐associated resistance was represented by *bla*_NDM‐1_, detected in 16 of 122 isolates (13.11%). The *bla*_NDM‐1_‐positive isolates were concentrated in four ST/capsule/O‐locus backgrounds rather than distributed uniformly across the cohort.

ST3805/KL8/OL2α.2 was the most frequent *bla*_NDM‐1_‐positive background (7/16, 43.75%). ST38/KL52/OL13 and ST15/KL112/OL2α.1 each accounted for four isolates (25.00%), and one isolate belonged to ST278/KL136/OL2α.2 (6.25%). All ST3805 and ST38 isolates were *bla*_NDM‐1_‐positive, whereas ST15 contained a smaller carbapenemase‐positive subset within the dominant lineage (Table 2).

**Table 2:** Genomic backgrounds of *bla*_NDM‐1_‐positive isolates.

Genomic background	No. of *bla*_NDM‐1_‐positive isolates	% of *bla*_NDM‐1_ subset	Interpretation
ST3805/KL8/OL2α.2	7/16	43.75	Most frequent *bla*_NDM‐1_‐positive background
ST38/KL52/OL13	4/16	25.00	Recurrent *bla*_NDM‐1_‐positive background
ST15/KL112/OL2α.1	4/16	25.00	*bla*_NDM‐1_‐positive subset within dominant ST15 lineage
ST278/KL136/OL2α.2	1/16	6.25	Singleton *bla*_NDM‐1_‐positive background

### 3.4 Virulence‐Associated Loci and Kleborate Virulence Scores

Yersiniabactin‐associated loci were identified in 48 isolates (39.34%) and aerobactin‐associated loci in 43 (35.25%); colibactin and *rmpADC* were not detected. Forty‐three isolates had a Kleborate virulence score ≥ 3, corresponding to aerobactin‐positive genomic profiles. This group was concentrated in ST15 (32/41, 78.05%) and ST48 (9/17, 52.94%). In contrast, ST3805 and ST38 were uniformly *bla*_NDM‐1_‐positive but had no isolates with virulence score ≥ 3. Because these scores were inferred from genomic loci and no phenotypic virulence data were available, they were interpreted as genotype‐based virulence profiles rather than confirmation of hypervirulence (Table 3).

**Table 3:** Virulence‐associated loci and Kleborate virulence score ≥ 3 by major sequence type.

Group/lineage	No. of isolates	Virulence score ≥ 3	Main virulence feature(s)	*bla*_NDM‐1_‐positive isolates	Porin‐associated resistance features	Main interpretation
Overall cohort	122	43/122 (35.25%)	*ybt*: 48/122 (39.34%); *iuc*: 43/122 (35.25%); *clb*: 0/122; *rmpADC*: 0/122	16/122 (13.11%)	63/122 (51.64%)	Virulence profile was dominated by siderophore‐associated loci; colibactin and *rmpADC*‐associated loci were absent.
ST15	41	32/41 (78.05%)	Aerobactin‐positive isolates with virulence score ≥ 3: 32/41 (78.05%)	4/41 (9.76%)	40/41 (97.56%)	Dominant lineage with frequent virulence score ≥ 3 profiles and a smaller *bla*_NDM‐1_‐positive subpopulation.
ST48	17	9/17 (52.94%)	Yersiniabactin‐positive: 17/17 (100%); aerobactin‐positive: 9/17 (52.94%)	0/17 (0.00%)	12/17 (70.59%)	Recurrent lineage with siderophore‐associated virulence but no *bla*_NDM‐1_‐positive isolates.
ST336	11	0/11 (0.00%)	Yersiniabactin‐positive: 11/11 (100%)	0/11 (0.00%)	0/11 (0.00%)	Lineage carried yersiniabactin but did not have a virulence score ≥ 3.
ST3805	7	0/7 (0.00%)	Yersiniabactin‐positive: 7/7 (100%)	7/7 (100%)	7/7 (100%)	Concentrated *bla*_NDM‐1_‐positive background with porin‐associated resistance features but no virulence score ≥ 3 profile.
ST711	7	0/7 (0.00%)	No virulence score ≥ 3 profile detected in this lineage	0/7 (0.00%)	0/7 (0.00%)	Recurrent lineage without high‐virulence‐score or *bla*_NDM‐1_‐positive profile in this dataset.
ST38	4	0/4 (0.00%)	No virulence score ≥ 3 profile detected in this lineage	4/4 (100%)	4/4 (100%)	Smaller *bla*_NDM‐1_‐positive background with porin‐associated resistance features but no virulence score ≥ 3 profile.

### 3.5 Capsular, O‐Antigen and Surface‐Locus Diversity

Capsule and O‐antigen locus prediction revealed lineage‐associated surface‐locus diversity. KL112 was the most frequent capsule locus (41/122, 33.61%), followed by KL62 (17/122, 13.93%), KL25 (11/122, 9.02%), KL54 (7/122, 5.74%), and KL8 (7/122, 5.74%). OL2α.1 (64/122, 52.46%) and OL2α.2 (34/122, 27.87%) together accounted for 80.33% of isolates; OL5, OL13, OL4, and OL3γ were less frequent. The predominant combined backgrounds were ST15/KL112/OL2α.1 (39/122, 31.97%), ST48/KL62/OL2α.1 (17/122, 13.93%), ST336/KL25/OL5 (11/122, 9.02%), ST3805/KL8/OL2α.2 (7/122, 5.74%) and ST711/KL54/OL2α.2 (7/122, 5.74%). The *bla*_NDM‐1_‐positive ST3805 and ST38 lineages were associated with distinct KL/O‐locus backgrounds (Table 4).

**Table 4:** Major sequence type, capsule‐locus, and O‐locus combinations in the 122‐isolate cohort.

Sequence type	Capsule locus	O‐locus	No. isolates	% of cohort	Interpretation
ST15	KL112	OL2α.1	39	31.97	Dominant surface‐locus background in the cohort
ST48	KL62	OL2α.1	17	13.93	Second most frequent ST/KL/O combination
ST336	KL25	OL5	11	9.02	Recurrent non‐ST15 genomic background
ST3805	KL8	OL2α.2	7	5.74	Principal *bla*_NDM‐1_‐positive background
ST711	KL54	OL2α.2	7	5.74	Recurrent OL2α.2‐associated lineage
ST17	KL122	OL2α.2	4	3.28	Minor recurrent lineage
ST22	KL9	OL2α.2	4	3.28	Small lineage with relatively low within‐ST SNP distances
ST38	KL52	OL13	4	3.28	*bla*_NDM‐1_‐positive background
ST278	KL136	OL2α.2	3	2.46	Included one *bla*_NDM‐1_‐positive isolate
ST397	KL158	OL2α.1	3	2.46	Minor recurrent lineage
ST13	KL3	OL2α.2	2	1.64	Low‐frequency background
ST25	KL2	OL2α.2	2	1.64	Low‐frequency background
ST29	KL19	OL2α.2	2	1.64	Low‐frequency background
ST37	KL118	OL13	2	1.64	Low‐frequency OL13 background
ST3806	KL2	OL2α.1	2	1.64	Low‐frequency background
ST437	KL36	OL4	2	1.64	Low‐frequency OL4 background
ST474	KL17	OL4	2	1.64	Low‐frequency OL4 background
ST15	KL112	OL2α.2	1	0.82	Minor surface‐locus variant within ST15
ST15	KL24	OL2α.1	1	0.82	Minor surface‐locus variant within ST15
ST2258	KL131	OL4	1	0.82	Singleton background

### 3.6 Plasmid Replicon Distribution

PlasmidFinder identified 32 replicon types. IncFIB(K)_1_Kpn3 was the most frequent (101/122, 82.79%), followed by IncHI1B_1_pNDM‐MAR (61/122, 50.00%), Col440II_1 and ColpVC_1 (50/122 each, 40.98%), IncFIB(pKPHS1)_1_pKPHS1 (47/122, 38.52%), repB(R1701)_1 (36/122, 29.51%), and IncFIB(Mar)_1_pNDM‐Mar (32/122, 26.23%). Replicon profiles were strongly lineage associated. ST15 commonly carried IncFIB(pKPHS1)_1_pKPHS1, IncFIB(K)_1_Kpn3, ColpVC_1, Col440II_1, and IncHI1B_1_pNDM‐MAR; IncFIB(K)_1_Kpn3 was present in all ST48, ST336 and ST711 isolates. All ST3805 isolates carried Col440II_1, IncHI2A_1, IncHI2_1, and repB(R1701)_1, whereas all ST38 isolates carried Col440I_1, IncA/C2_1, IncFIB(K)_1_Kpn3, IncFIB(pQil)_1_pQil, and IncFII_1_pKP91. These replicon‐marker profiles do not resolve complete plasmid structures or demonstrate direct linkage between a detected replicon and *bla*_NDM‐1_ (Table 5).

**Table 5:** Plasmid replicon distribution by cohort and major sequence‐type backgrounds.

Category	Major replicon pattern	No. positive isolates	Interpretation
Overall cohort	IncFIB(K)_1_Kpn3	101/122 (82.79%)	Most frequent plasmid replicon
Overall cohort	IncHI1B_1_pNDM‐MAR	61/122 (50.00%)	Common IncHI1B‐associated replicon signature
Overall cohort	Col440II_1; ColpVC_1	50/122 each (40.98%)	Frequent Col‐type replicon signatures
ST15	IncFIB(pKPHS1)_1_pKPHS1; IncFIB(K)_1_Kpn3; ColpVC_1	40/41; 37/41; 36/41	Dominant ST15 plasmid‐replicon background
ST48	IncFIB(K)_1_Kpn3	17/17 (100.00%)	Universal in ST48
ST3805	Col440II_1; IncHI2A_1; IncHI2_1; repB(R1701)_1	7/7 each (100.00%)	Structured replicon background in *bla*_NDM‐1_‐positive ST3805
ST38	Col440I_1; IncA/C2_1; IncFIB(K)_1_Kpn3; IncFIB(pQil)_1_pQil; IncFII_1_pKP91	4/4 each (100.00%)	Structured replicon background in *bla*_NDM‐1_‐positive ST38

### 3.7 Reference‐Based SNP Diversity and Within‐Lineage Relatedness

**Figure 2:**
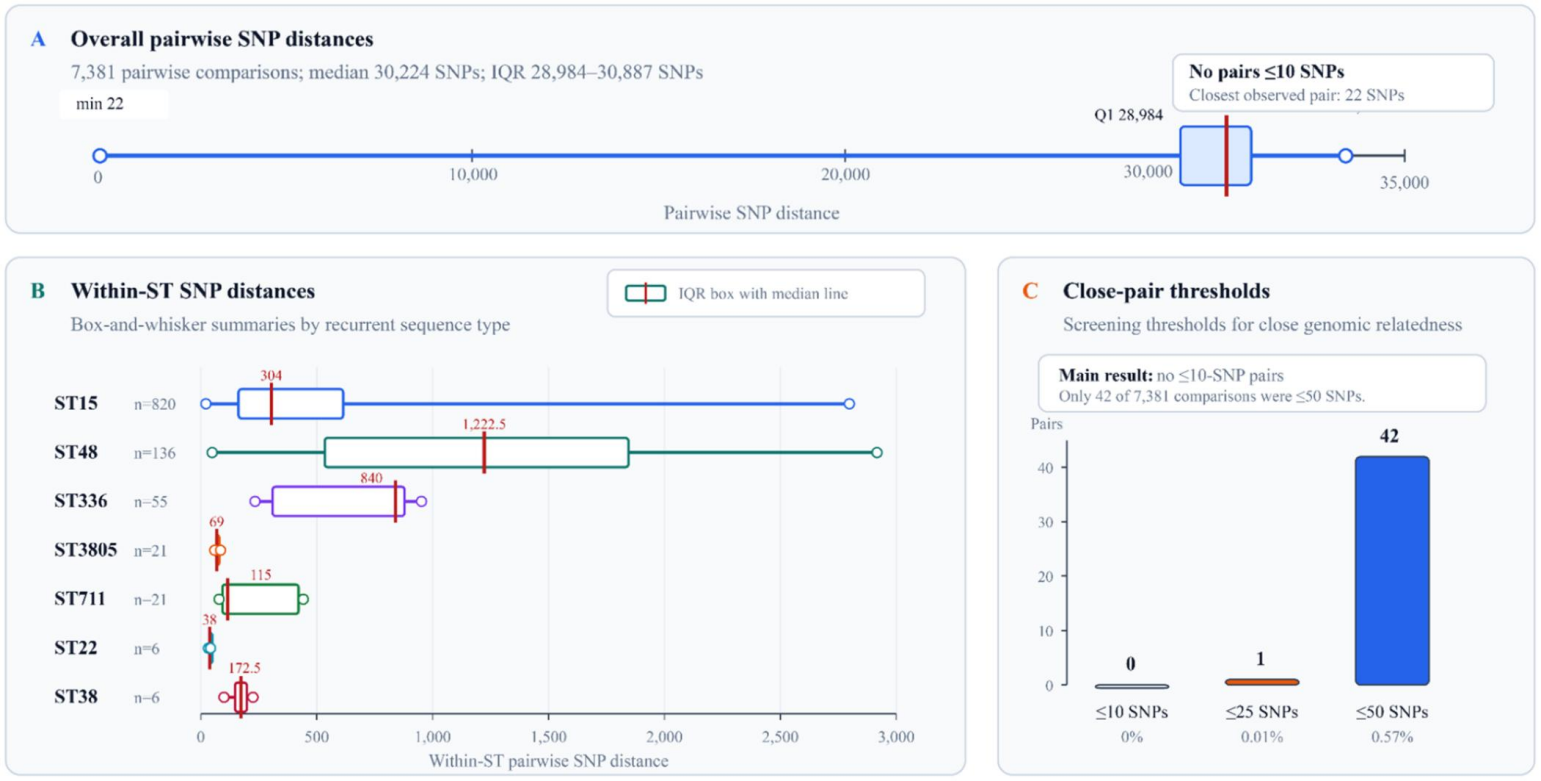
Reference‐based SNP diversity and within‐lineage relatedness. (A) Five‐number summary of 7381 pairwise SNP distances calculated from 169,220 high‐confidence biallelic SNP sites. (B) Within‐sequence‐type five‐number summaries for recurrent lineages. (C) Exact counts of isolate pairs at ≤ 10, ≤ 25, and ≤ 50 SNP descriptive screening thresholds. Threshold categories were used to summarize genomic proximity and were not treated as universal transmission cutoffs.

Reference‐based cohort analysis identified 169,220 high‐confidence biallelic SNP sites across the 122 isolates (Figure 2). The 7381 pairwise comparisons had a median distance of 30,224 SNPs (IQR, 28,984–30,887; range, 22–33,422). No pair differed by ≤ 10 SNPs, one pair differed by ≤ 25 SNPs and 42 pairs differed by ≤ 50 SNPs. These descriptive thresholds therefore identified only a small subset of relatively close comparisons and did not support a single outbreak‐like population. Within‐lineage distances varied substantially: ST15 had a median of 304 SNPs (range, 22–2798; 820 pairs), ST48 1222.5 (49–2917; 136 pairs), ST336 840 (234–952; 55 pairs), ST3805 69 (60–85; 21 pairs), ST711 115 (79–442; 21 pairs), ST22 38 (33–42; six pairs) and ST38 172.5 (100–225; six pairs). Overall and within‐ST summaries are provided in Table 6.

**Table 6:** Reference‐based SNP diversity and pairwise genomic‐distance summary for the 122‐isolate cohort.

Metric	Value	Interpretation
No. isolates	122	Final cohort size
High‐confidence biallelic SNP sites	169,220	Cohort‐level reference‐based SNP set
Pairwise comparisons	7381	All isolate‐pair comparisons
Minimum pairwise SNP distance	22	Closest isolate pair in the cohort
Q1 pairwise SNP distance	28,984	Lower quartile
Median pairwise SNP distance	30,224	Overall population‐level diversity
Q3 pairwise SNP distance	30,887	Upper quartile
Maximum pairwise SNP distance	33,422	Most divergent isolate pair
Pairs ≤ 10 SNPs	0	No pairs met a very‐close relatedness threshold
Pairs ≤ 25 SNPs	1	Only one close pair at this threshold
Pairs ≤ 50 SNPs	42	Close relatedness restricted to a small subset of comparisons

Overall, the SNP results show broad diversity across the cohort and narrower relatedness within selected sequence types. They should be interpreted as descriptive reference‐based genomic distances rather than evidence of direct transmission.

**Table 7:** Integrated genomic profiles of major lineage‐associated backgrounds.

Lineage/background	No. isolates	Surface locus	AMR/carbapenemase profile	Virulence profile	Plasmid/SNP interpretation
ST15	41	Mainly KL112/OL2α.1	*bla*_CTX‐M‐15_ common; 4 *bla*_NDM‐1_‐positive isolates	32/41 isolates with virulence score ≥ 3	Dominant MDR and virulence‐associated lineage with several close within‐ST pairs
ST3805	7	KL8/OL2α.2	7/7 *bla*_NDM‐1_‐positive; porin‐associated features present	No isolates with virulence score ≥ 3	Concentrated carbapenemase‐positive background with narrow within‐ST SNP range
ST38	4	KL52/OL13	4/4 *bla*_NDM‐1_‐positive; porin‐associated features present	No isolates with virulence score ≥ 3	Small carbapenemase‐positive lineage with distinct plasmid‐replicon profile
ST48	17	KL62/OL2α.1	No *bla*_NDM‐1_‐positive isolates detected	9/17 isolates with virulence score ≥ 3; *ybt* in 17/17	Virulence‐associated lineage distinct from principal carbapenemase‐positive backgrounds
ST336/ST711/ST22	11/7/4	KL25/OL5; KL54/OL2α.2; KL9/OL2α.2	No *bla*_NDM‐1_‐positive isolates detected in these recurrent groups	Variable siderophore‐associated profiles	Additional recurrent lineage‐specific backgrounds

### 3.8 Integrated Lineage‐Associated Genomic Profiles

**Figure 3:**
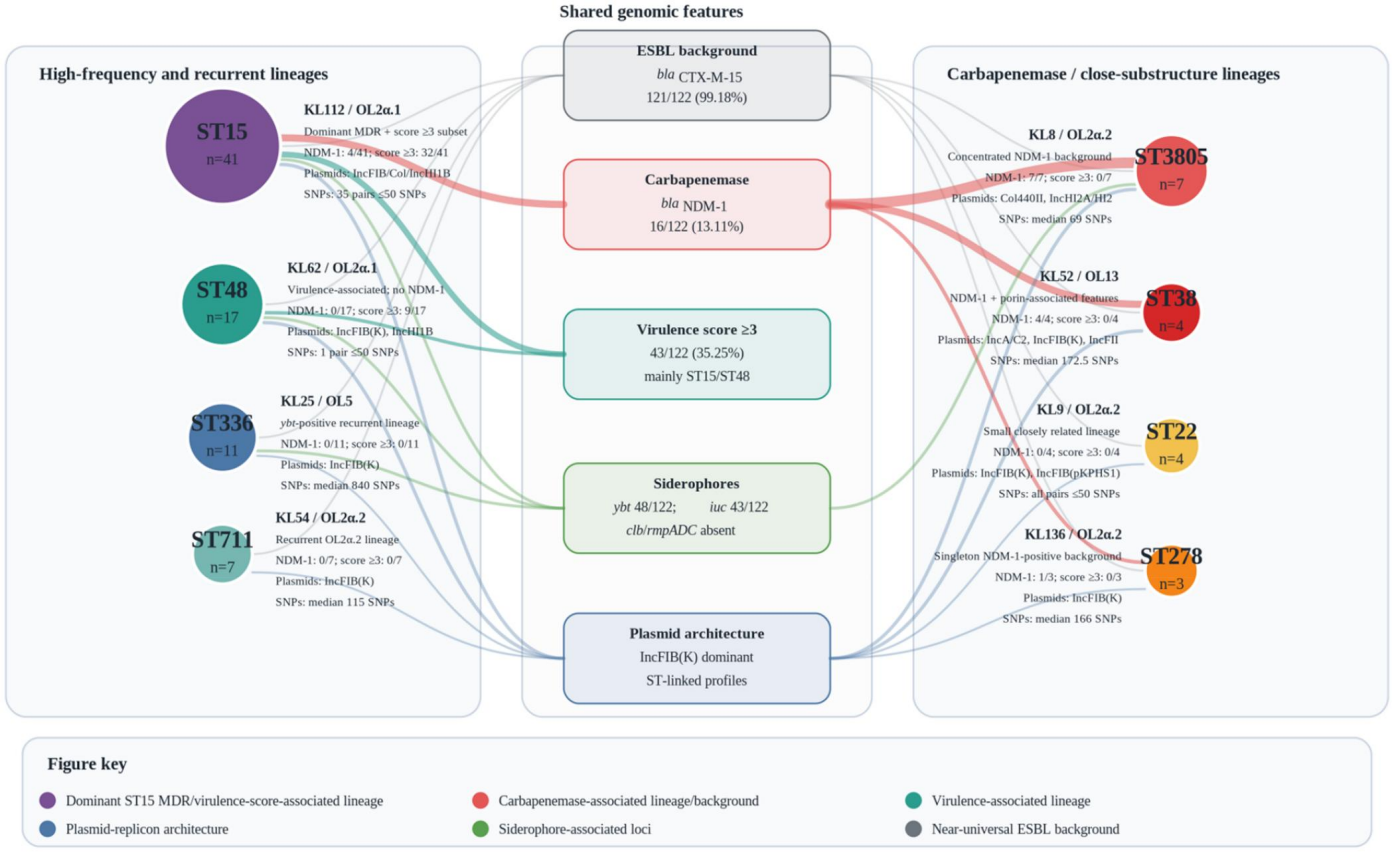
Integrated genomic profiles of major *Klebsiella pneumoniae* lineages. Cohort‐level schematic linking recurrent sequence types with their dominant capsule/O‐antigen backgrounds, *bla*_NDM‐1_ distribution, Kleborate virulence score ≥ 3, siderophore loci, plasmid‐replicon marker profiles, and within‐lineage SNP summaries. Nodes and links summarize validated results reported in Tables 2, 3, 4, 5, 6, 7; link thickness is illustrative and does not represent a formal effect size or statistical association.

Integrated analysis identified several lineage‐associated genomic profiles (Figure 3; Table 7). ST15 was the dominant background (41/122), was mainly associated with KL112/OL2α.1, included 32 isolates with Kleborate virulence score ≥ 3 and four *bla*_NDM‐1_‐positive isolates, and carried a broad IncFIB/Col/IncHI1B replicon‐marker profile. ST3805 and ST38 represented concentrated *bla*_NDM‐1_‐positive backgrounds with distinct surface loci and replicon combinations; their median within‐ST distances were 69 and 172.5 SNPs, respectively. ST48 (17/122) was associated with KL62/OL2α.1, universal yersiniabactin carriage and nine isolates with virulence score ≥ 3, but no *bla*_NDM‐1_‐positive isolate. ST336, ST711, and ST22 represented additional recurrent backgrounds with characteristic surface‐locus and SNP‐distance patterns. Collectively, resistance, virulence‐associated loci, surface antigens, and plasmid‐replicon markers were strongly structured by lineage rather than distributed uniformly across the cohort.

## 4 Discussion

This study provides an integrated genomic analysis of 122 publicly available human clinical *K. pneumoniae* WGS datasets from Pakistan. The cohort contained 23 sequence types and substantial pairwise SNP diversity, indicating multiple co‐circulating lineages rather than a single clonal expansion. ST15 was dominant, while ST48, ST336, ST711, and ST3805 formed additional recurrent backgrounds. The integrated analysis further showed that resistance determinants, virulence‐associated loci, surface antigens, and plasmid‐replicon markers were strongly structured by lineage. The predominance of ST15 is consistent with reports describing regional expansion and healthcare‐associated dissemination of this multidrug‐resistant high‐risk lineage (Zhou et al. 2016).

The resistance profile was dominated by *bla*_CTX‐M‐15_, detected in 121 of 122 isolates, together with frequent *bla*_VEB‐5_, plasmid‐mediated quinolone‐resistance determinants, and predicted porin alterations. These findings are consistent with reports of ESBL‐producing *K. pneumoniae* and other resistant Gram‐negative pathogens in Pakistan (Khan et al. 2025; Idrees et al. 2023). *bla*_NDM‐1_ occurred in 16 isolates and was concentrated in four ST/KL/OL backgrounds, consistent with the regional importance and mobility of NDM‐associated resistance (Walsh 2011; Qamar et al. 2025; Lascols et al. 2023). These results indicate that carbapenemase carriage was not limited to one clonal lineage.

Plasmid‐replicon screening added context to the resistance findings. IncFIB(K)_1_Kpn3 was the most frequent marker, and ST15, ST3805, and ST38 showed distinct replicon combinations. Plasmids are established vehicles for quinolone‐resistance and β‐lactamase determinants (Jacoby et al. 2014), and studies from Pakistan have documented diverse plasmid backbones carrying clinically important resistance genes (Qamar et al. 2025; Lascols et al. 2023). However, short‐read replicon detection does not establish complete plasmid structure or prove that a particular resistance gene is located on a detected replicon; the present results should therefore be interpreted as lineage‐associated plasmid‐background signals.

Virulence‐associated loci were also lineage‐structured. Yersiniabactin and aerobactin were detected in 39.34% and 35.25% of isolates, respectively, whereas colibactin and *rmpADC* were absent. Kleborate virulence scores ≥ 3 were concentrated in ST15 and ST48, while the principal *bla*_NDM‐1_‐positive ST3805 and ST38 backgrounds had lower scores. This pattern suggests accumulation of selected siderophore‐associated loci in multidrug‐resistant backgrounds rather than the broader classical hypervirulence‐associated genotype associated with aerobactin, colibactin, and *rmp*‐linked hypermucoviscosity (Mendes et al. 2023; Arena et al. 2022; Najafian et al. 2025). Genomic virulence scores alone, however, do not establish a hypervirulent phenotype.

The SNP‐distance analysis resolves a key feature of the cohort. The overall median pairwise distance was 30,224 SNPs, and no pair differed by ≤ 10 SNPs, demonstrating broad genomic diversity. Some recurrent STs contained relatively close comparisons, but fixed SNP thresholds are sensitive to the lineage, variant‐calling pipeline, reference choice, and epidemiological setting (Luterbach et al. 2023). Accordingly, the ≤ 10, ≤ 25, and ≤ 50 SNP categories used here are descriptive screening thresholds and should not be interpreted as evidence of transmission without temporal and epidemiological linkage.

Phenotypic antimicrobial‐susceptibility data were unavailable, so treatment implications cannot be inferred directly from genotype. Nevertheless, the near‐universal ESBL background, frequent PMQR determinants, and lineage‐structured *bla*_NDM‐1_ carriage underscore the need for local phenotypic surveillance, rapid carbapenemase detection, and susceptibility‐guided therapy. From a public health perspective, the findings support genomic surveillance approaches that integrate sequence type, resistance and virulence determinants, surface loci, plasmid‐replicon markers and genomic relatedness.

## 5 Study Limitations

This study has several limitations. First, phenotypic antimicrobial‐susceptibility testing was not available, precluding direct genotype–phenotype assessment. Second, detailed patient‐level clinical, temporal, and outcome metadata were incomplete, limiting epidemiological inference. Third, the datasets originated from a single public BioProject and may not represent the national diversity of clinical *K. pneumoniae* in Pakistan. Fourth, PlasmidFinder analysis of short‐read assemblies identifies replicon markers but does not resolve complete plasmids, gene linkage or transfer units. Finally, SNP distances were derived from a single‐reference framework without explicit recombination masking and therefore represent uncorrected reference‐based genomic differences rather than a transmission‐optimized core‐genome analysis.

## 6 Future Directions

Future work should combine prospective, geographically representative sampling with phenotypic antimicrobial‐susceptibility testing, standardized clinical metadata, and patient outcomes. Long‐read or hybrid sequencing would resolve complete plasmids and establish the physical linkage of *bla*_NDM‐1_, ESBL genes, virulence loci, and replicon backbones. Recombination‐aware phylogenetic analysis and longitudinal sampling would help determine whether the ST15, ST3805, ST38, and ST48 backgrounds represent persistent local lineages, repeated introductions, or broader regional transmission networks.

## 7 Conclusions

The 122 clinical *K. pneumoniae* datasets comprised multiple co‐circulating genomic backgrounds rather than a single clonal expansion. ST15 was dominant and combined extensive resistance determinants, frequent Kleborate virulence scores ≥ 3 and broad plasmid‐replicon carriage. *bla*_NDM‐1_‐positive isolates were concentrated in ST3805, ST38, ST15, and ST278 backgrounds with distinct capsule, O‐antigen and replicon profiles. These findings provide a dataset‐specific genomic reference point for multidrug‐resistant clinical *K. pneumoniae* from Pakistan and support integrated genomic, phenotypic, and epidemiological surveillance.

## Author Contributions

**Hakeem Zada:** conceptualization, visualization, validation, investigation, writing – review and editing. **Muhammad Qasim:** investigation, writing – original draft, methodology, data curation, project administration. **Mubashir Hussain:** investigation, writing – original draft, conceptualization, validation, resources. **Abdul Rehman:** investigation, writing – original draft, conceptualization, validation, resources. **Hassan Naveed:** funding acquisition, investigation, validation, methodology, formal analysis. **Noor Bahadar:** writing – original draft, conceptualization, investigation, writing – review and editing, methodology. **Najibullah Anwari:** software, formal analysis, project administration, resources, visualization, supervision, writing – review and editing.

## Funding

The authors have nothing to report.

## Ethics Statement

This study analyzed publicly available, de‐identified sequence data.

## Consent

The authors have nothing to report.

## Conflicts of Interest

The authors declare no conflicts of interest.

## Data Availability

The raw sequencing data analyzed in this study are publicly available in the NCBI Sequence Read Archive under BioProject PRJEB6574. Additional analysis details are available from the corresponding author on reasonable request. The data that support the findings of this study are available in NCBI Bioproject at https://www.ncbi.nlm.nih.gov/bioproject/?term=PRJEB6574, reference number PRJEB6574. These data were derived from the following resources available in the public domain.
